# Preventive governance for patient‐centred medical AI

**DOI:** 10.1002/ctm2.70872

**Published:** 2026-10-13

**Authors:** Linghua Yu

**Affiliations:** ^1^ The First Hospital of Jiaxing Zhejiang Province People's Republic of China

## 1 THE GOVERNANCE PROBLEM IS CHANGING

Medical artificial intelligence (AI) is advancing from information provision and clinical assistance towards decision support and expanding clinical authority. Its reach now extends into diagnosis, treatment planning, monitoring, and patient management. Governance concerns have accordingly broadened from model performance alone to decision authority, accountability, meaningful human oversight, patient autonomy and health equity.^1^, ^2^, ^3^ Recent work on AI‐driven clinical consultations and autonomous agents indicates that these questions have shifted from hypothetical scenarios to emerging clinical realities.^4^, ^5^

One question threads through these discussions: what happens when something goes wrong? Who made the decision, who should have intervened, and who is accountable? These questions remain essential.^5^, ^6^ Autonomous systems, however, raise a complementary problem. What should be done before something goes wrong? Figure 1

**FIGURE 1:**
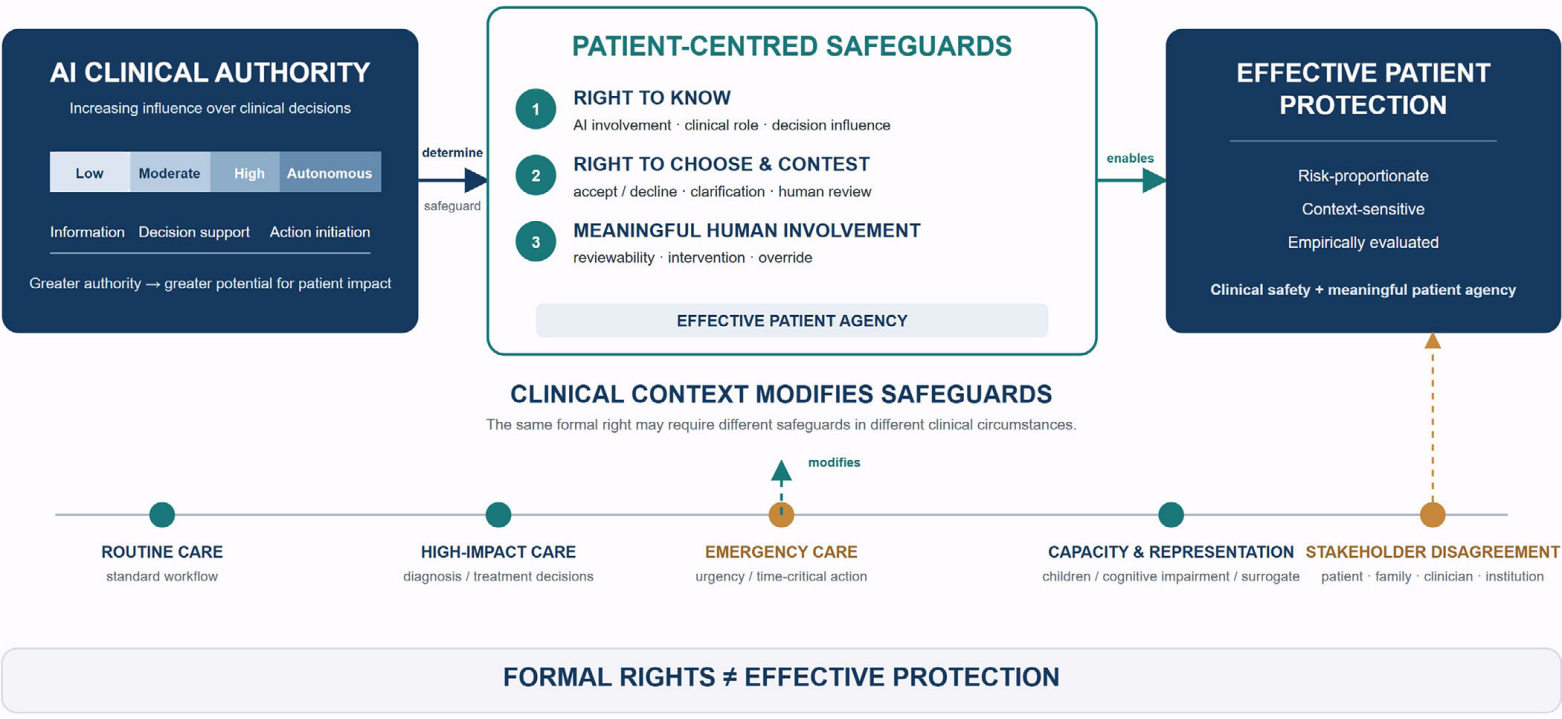
Patient‐centred governance across clinical contexts. Patient‐centred safeguards must strengthen as AI clinical authority increases: the right to know how AI participates in care, to choose and contest that involvement, and to meaningful human involvement through reviewability, intervenability and override. Clinical context determines whether these safeguards protect in practice or remain merely formal.

Medical AI governance should therefore move beyond allocating responsibility after harm toward anticipating patient risk and preventing avoidable harm before it occurs.

## 2 PATIENT AGENCY AS A GOVERNANCE LAYER

Patient autonomy should be treated not only as an ethical principle at the point of consent, but as a potential safety mechanism throughout AI‐mediated care. Growing recognition of patient rights over health data and AI governance reinforces the case for positioning patients more explicitly within the governance architecture.^3^, ^7^

This places patient agency alongside technical performance, institutional accountability and human oversight as a component of clinical safety. It raises three practical questions. Can patients know that AI is involved in their care? Can they choose and contest its role? And can they reach meaningful human intervention when it matters most? These questions translate into three complementary safeguards.

## 3 THREE PATIENT‐CENTRED SAFEGUARDS

### 3.1 Right to Know

Patients should know whether AI meaningfully participates in their care, where it enters the clinical pathway, what decisions it can influence, and which remain under human control. Disclosure should therefore describe what AI is doing in a patient's care, not merely that ‘AI is used’. The distinction matters more as AI moves from documentation into diagnosis and treatment‐related functions, and sharpens where ambient systems embed AI within routine clinical communication. Such systems raise questions of awareness, consent, and trust before patients can pose them.^8^

### 3.2 Right to Choose and Contest

Where legally and clinically feasible, patients should be able to accept or decline AI involvement and to reach a clinically reasonable non‐AI pathway. Refusal of AI‐supported diagnosis and treatment planning has an established ethical basis, but it must be read against the context in which the choice is offered.^9^ Contestation extends beyond the initial decision: patients should be able to question an AI‐mediated recommendation, request clarification, and obtain human review or a further clinical assessment.

Yet information is not agency, and consent is not contestability. A right to refuse is meaningful only when a clinically reasonable alternative exists.

### 3.3 Right to Meaningful Human Involvement

Human oversight should not be equated with the mere presence of a clinician in the loop. Meaningful involvement requires reviewability, intervenability and override: clinicians need enough information and opportunity to understand an AI‐mediated decision, to intervene when necessary, and to alter, suspend, or reverse its consequences. Meaningful medical AI oversight has likewise been argued to depend on epistemic capacity, cognitive space, decisional authority and intervention effectiveness.^2^ Knowing, choosing and reaching meaningful human intervention together form a prospective safety barrier around AI‐mediated care.

## 4 WHERE THESE RIGHTS MAY FAIL

These safeguards cannot be implemented through a single universal consent procedure. In life‐threatening emergencies, extensive pre‐procedure disclosure or choice may itself delay time‐critical intervention. Governance must therefore consider risk‐adaptive exceptions, rather than assuming that consent always precedes action or that clinical safety automatically overrides autonomy.

Decision‐making capacity imposes a different constraint. Unconscious patients, people with cognitive impairment or delirium, and children may be unable to exercise these rights independently, and decisions may then involve interactions among patient preference, surrogate authority, and clinical necessity. In paediatric care, adolescents may be familiar with and receptive to AI while parents or guardians differ in their understanding or acceptance.

A further boundary condition concerns who is positioned to claim these rights. Disagreement may arise among patients, family members, clinicians, and institutions, and the operative question becomes not who can refuse AI but who decides when legitimate stakeholders disagree about its involvement. Unequal digital literacy and access cut across the same problem: formally identical rights are least exercisable by the patients who stand to benefit most, and unequal capability may translate into unequal benefit.^10^

## 5 FROM FORMAL RIGHTS TO EFFECTIVE PATIENT PROTECTION

The central challenge is that formal rights do not necessarily produce effective protection. Disclosure can be issued without being understood, and oversight can be convened without the capacity to intervene. A right of refusal fares no better when there is no alternative in favour of which to refuse.

Governance should therefore ask not whether a safeguard exists, but whether it works under the conditions in which it must protect the patient.

The level of protection required should scale with AI authority, clinical impact, potential harm, reversibility, urgency and decision‐making capacity. The stakes rise as autonomous and agentic systems widen the range of actions AI may initiate within clinical workflows.^4^, ^5^ Patient‐centred governance should accordingly be risk‐proportionate, context‐sensitive and empirically evaluated.

## 6 FUTURE OUTLOOK

Future governance must anticipate the patient risks of expanding AI clinical authority and adapt safeguards to clinical context. Whether those safeguards protect patients’ ability to know, choose, contest and obtain meaningful human care is ultimately empirical, settled by whether safety and autonomy improve in practice.

This preventive perspective complements accountability rather than replacing it. The task is to connect the emerging focus on AI authority and responsibility with a corresponding framework for patient agency and protection.^6^, ^7^ The goal is not only to establish who is responsible after harm, but to prevent avoidable harm while preserving meaningful patient agency before harm occurs.

## AUTHOR CONTRIBUTIONS

The author conceived the manuscript, conducted the literature review and analysis, drafted and revised the manuscript, and approved the final version. The author is accountable for all aspects of the work.

## CONFLICT OF INTEREST STATEMENT

The author declares no conflicts of interest.

## ETHICS STATEMENT

Not applicable. This article does not involve human participants, animals, or identifiable personal data.
